# Chokehold with ‘rear naked choke’ and delayed post-hypoxic leukoencephalopathy: a new form of assault in Mexico City

**DOI:** 10.1007/s00701-025-06601-9

**Published:** 2025-07-19

**Authors:** Carlos Castillo-Rangel, Cristofer Zarate-Calderon, Carlos Castillo-Soriano, Karla Aketzalli Hernández-Contreras, Gerardo Marín-Márquez

**Affiliations:** 1 Department of Neurosurgery, Hospital Regional “1° de Octubre”, Institute of Social Security and Services for State Workers (ISSSTE), Mexico City, 07300 Mexico; 2 Department of Biophysics, Brain Research Institute , Universidad Veracruzana, Xalapa, Veracruz 91190 Mexico; 3 Tecnológico de Monterrey, Villa High and Villa Educativa, Monterrey, Mexico; 4 Neural Dynamics and Modulation Lab, Cleveland Clinic, 9500 Euclid Avenue, Cleveland, OH 44196 USA; 5 Physical Education, Recreation and Sport Department, Instituto Universitario Veracruzano, Banderilla, Veracruz, México

**Keywords:** Delayed posthypoxic leukoencephalopathy, Hypoxic-ischemic encephalopathy, White matter, Brain biopsy, Strangulation

## Abstract

In Mexico City, a 49-year-old man underwent strangulation, which led to hypoxic-ischemic encephalopathy (HIE) and subsequently to delayed post-hypoxic leukoencephalopathy (DPHL), a rare demyelinating condition. Following the attack, he exhibited aphasia, dysphagia, and other neuropsychiatric symptoms that progressed to dementia. Imaging and brain biopsy analyses disclosed extensive ischemic damage and reactive gliosis. This case underscores the link between strangulation, acute HIE, and the subsequent development of DPHL, as well as an interpretation of the physiological implications of DPHL due to strangulation.

## Introduction

Hypoxic-ischemic encephalopathy (HIE) is a cerebral event where the oxygen supply (hypoxia) and blood flow (ischemia) are insufficient, leading to neural cell death and cerebral edema. Clinically, HIE presents with neurological signs within 24 h after the hypoxic event and varies in severity. At the cellular level, it involves energy failure, excitotoxicity, oxidative stress, and apoptosis, resulting in irreversible damage if anoxia persists for more than a few minutes [1, 6, 8].

On the other hand, delayed post-hypoxic leukoencephalopathy (DPHL) is a rare cerebral demyelinating syndrome, also known as delayed post-hypoxic encephalopathy (DPHE). It often appears after carbon monoxide poisoning; however, it has also been reported as a secondary consequence of various conditions such as hypoglycemia, strangulation, after cardiopulmonary resuscitation maneuvers, and as a complication of general anesthesia in surgery, as well as in self-poisoning with psychotropic drugs and toxins. It is characterized by the acute onset of neuropsychiatric symptoms days or weeks after the apparent recovery from a coma following a prolonged period of cerebral hypoxia [5, 7, 16].

## Clinical history

A 49-year-old male patient presents. No significant family medical history. Tobacco use: 5 cigarettes a day since the age of 18 (Smoking Index 7). Social alcohol consumption: 4 standard units per week for 18 years. No relevant chronic degenerative history.

The patient was assaulted on February 26, 2022: while walking through the streets of Mexico City, he reports that a person took him by surprise from behind and strangled him (applying a strangulation technique called"Rear Naked Choke"[RNC]), while two other people lifted his feet, causing him to lose consciousness within seconds. Minutes later, the patient woke up and decided to go home.

On February 27, he presented signs of motor aphasia and dysphagia to solids. No alteration in language comprehension, experiencing paresis in the lower limbs, leading to hospitalization, from which he fled the following day (February 28, 2022), with difficulties due to a right ankle sprain.

By March 2, the patient showed favorable clinical evolution, with a resolution of motor aphasia. However, he demonstrated an inability to remember recent events. Twenty-six days later, neurological integrity was reported. One month after the event, he developed sensory aphasia and short-term memory impairment, as well as delusional thoughts and prosopagnosia. He was discharged from the Mexican Institute of Social Security (IMSS) on March 31.

In the following days, further deterioration of cortical functions was observed, including short-term memory loss and gait disturbances, with a flexed trunk posture, rapid, short tiptoe steps, and balance alterations. He also experienced progressive language deterioration, losing the ability to recognize the names of his relatives (prosopagnosia vs. anomia) and losing bladder and bowel control. Additionally, spatial disorientation was recorded, along with myoclonus in the left hemibody, especially at night.

**Fig. 1:**
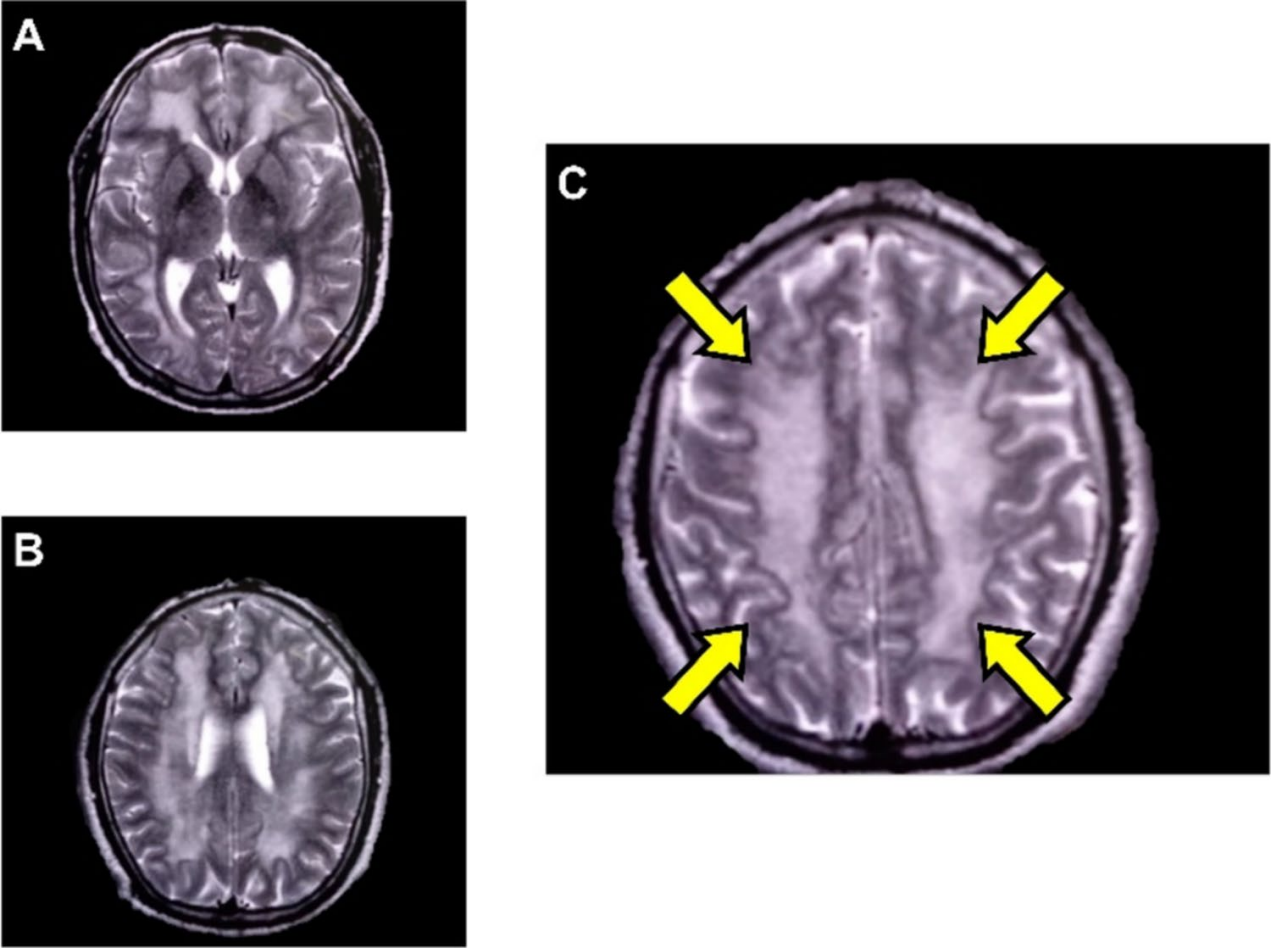
MRI showing extensive ischemic-type lesions in the white matter of the semi-oval centers **A**) Deep centers, **B**) High centers & **C**) Medial centers, evidenced by hyperintensity on diffusion, suggesting leukoencephalopathy

Treatment with risperidone 2 mg/24 h and lamotrigine 25 mg/24 h was initiated without improvement. The patient continued to deteriorate functionally, progressing to total dependence on basic daily activities. On April 14, magnetic resonance imaging (MRI) (magnetic resonance angiography) (Fig. 1 A, B & C) was performed, revealing extensive ischemic-type lesions in the white matter of the semi-oval centers due to hyperintensity on diffusion, suggesting DPHL of undetermined etiology.

On April 27, 2022, a diagnosis of rapidly progressive dementia was established, and hospitalization was decided for a brain biopsy.

Clinically, the patient presented with global aphasia, 3 mm pupil diameter, hyporeflexia, present conjunctival reflex, cylindrical neck, without murmurs or jugular engorgement, rhythmic heart sounds of adequate intensity, reflexes +/+ + + + in the left upper limb, and in the right lower limb, negative Babinski, negative Brudzinski and Kernig signs, negative Schaefer and Oppenheim signs.

The right frontal brain biopsy showed reactive gliosis, segmental loss of neurons, clusters of red neurons, edema, and spongiosis in the white matter. There was vascular congestion, focal hemorrhage, and perivascular lymphocytic inflammatory infiltrate. PAS staining highlighted areas of spongiosis, and Masson staining was positive in vascular walls. The diagnosis concluded with acute and chronic HIE with extensive gliosis.

Medical management was initiated with phenytoin 100 mg/8 h, lamotrigine 100 mg/12 h, and 1/2 tablet carbidopa-levodopa 250 mg/25 mg every 8 h.

**Fig. 2:**
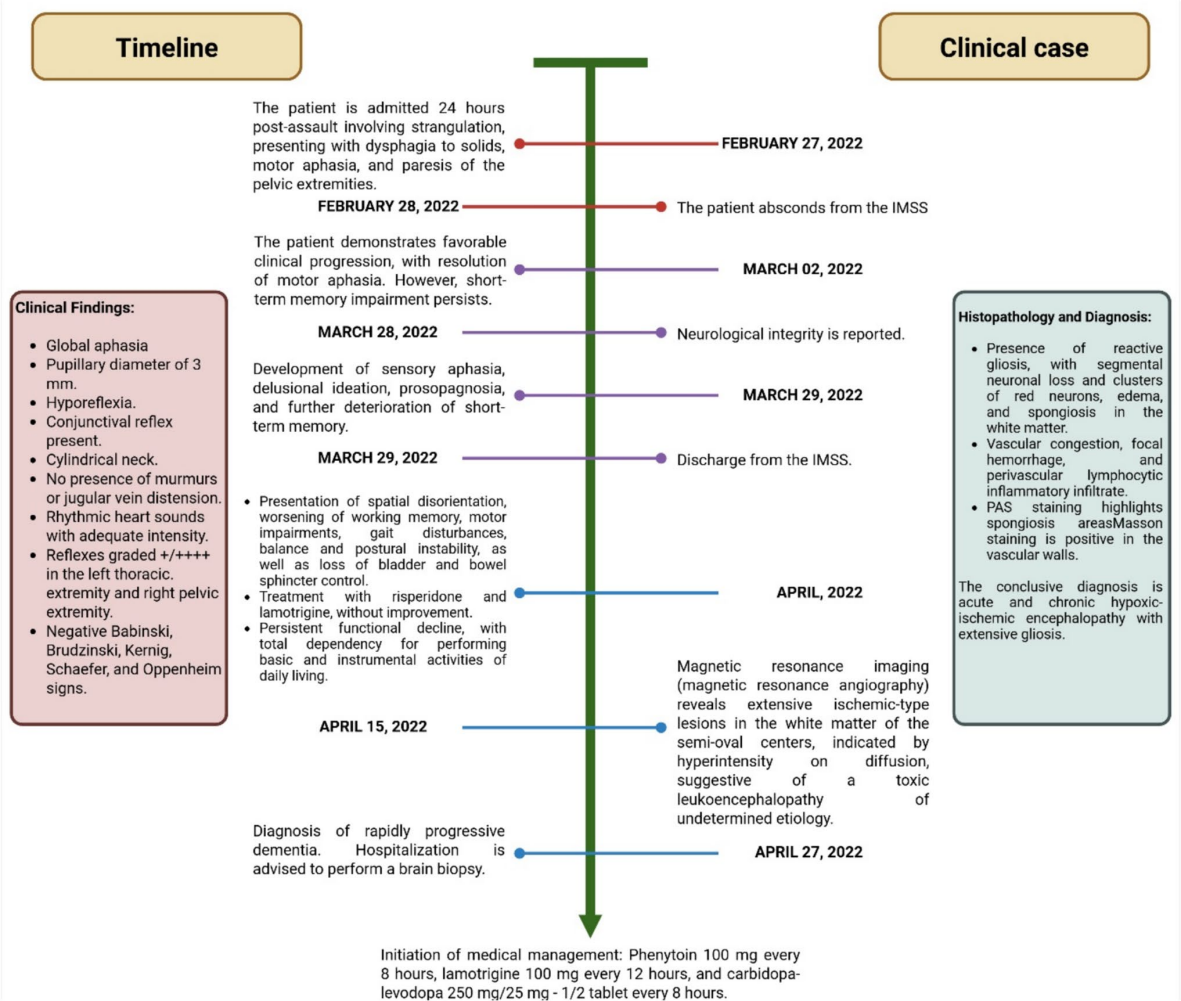
Timeline of the clinical case. This figure displays the main aspects of the patient's medical history

The patient's evolution can be observed in Fig. 2.

## Discussion

DPHL is a rare cerebral demyelinating syndrome characterized by the acute onset of neuropsychiatric symptoms days or weeks after the apparent recovery following a period of prolonged cerebral hypoxia [7, 16]. While the results of imaging studies initially suggested a possible HIE, the main characteristics of this pathology were omitted since it typically presents neurological symptoms within the first 24 h. The appearance of neuropsychiatric symptoms weeks after the initial strangulation episode is an essential indicator of the disease, as both clinical studies and the biopsy suggest a transition from acute brain injury to a prolonged and progressive pathological process.

## Trachea or carotids? How do we know strangulation is the etiology of DPHL?

The mechanism of RNC focuses on applying pressure around the neck. Unlike other strangulation techniques, it aims to cut off blood flow, explicitly targeting the carotid arteries, causing a decrease or complete cessation of oxygen supply to the brain. If prolonged, this can result in brain damage [2, 9]. Therefore, HIE occurs when brain tissue is damaged due to hypoxia and nutrient ischemia [6, 8].

The initial onset of symptoms such as motor aphasia and dysphagia suggests an acute HIE event, indicating a significant interruption of cerebral blood flow, most likely due to carotid compression. On the other hand, the diagnosis following the biopsy, DPHL, was determined by clinical characteristics such as progressive deterioration and the appearance of symptoms (sensory aphasia and memory impairment) weeks after the event, suggesting the evolution of the initial injury [9].

In the context of strangulation and considering the findings from the MRI and brain biopsy, it seems reasonable to attribute the cause of DPHL to the initial interruption of cerebral blood flow due to carotid compression. Finally, there is no report in the clinical history of dysphonia, anterior neck pain, or respiratory difficulty, which could suggest direct trauma to the trachea. If the trachea had been the main target of the assault, we would expect to see symptoms related to airway obstruction or tracheal damage, thus ruling out airway obstructions.

## Pathophysiology

**Fig. 3:**
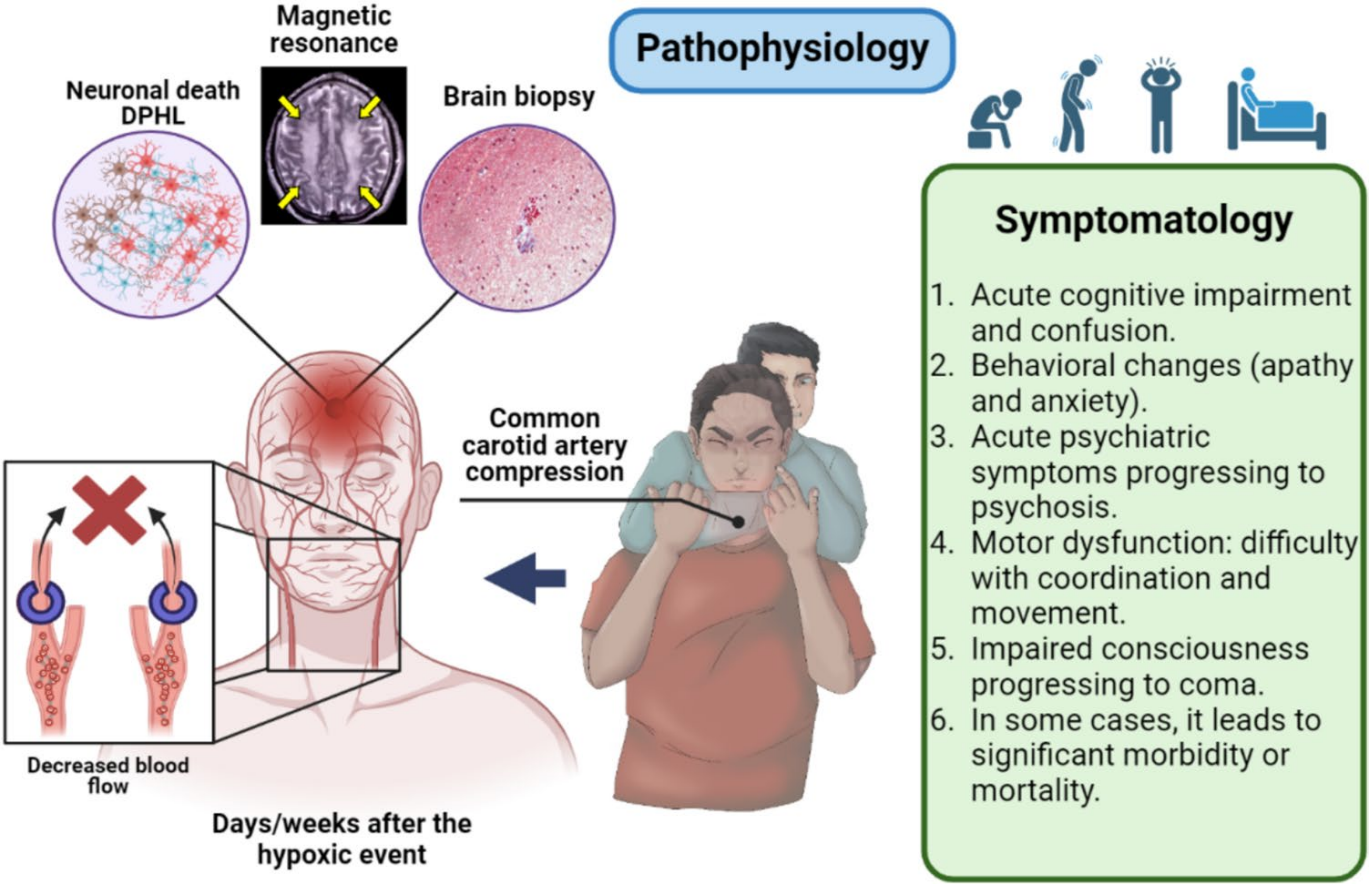
Illustration of potential aspects involved in DPHL (pathophysiology) and its main symptoms

The pathophysiology of DPHL due to strangulation (as in the case of RNC) is complex due to multiple pathological mechanisms. The primary biomechanism of RNC is to drastically reduce cerebral blood flow, diminishing oxygen and nutrient supply and resulting in HIE. These changes cause cellular damage through the excessive release of excitatory neurotransmitters, increasing intracellular calcium and activating destructive enzymes [12–14]. Simultaneously, hypoxia induces oxidative stress and free radical formation, exacerbating brain damage [4]. During blood reperfusion, further damage occurs through free radical production and inflammation [3]. Gliosis and potentially demyelination can be observed, reflecting progressive damage [9, 11]. Strangulation with carotid occlusion likely led to acute HIE, triggering pathological and physiological changes that progressed toward DPHL with delayed neurological deterioration. Symptoms can manifest days, weeks, or months after the hypoxic event, reflecting delayed brain damage. In this case, cerebral hypoxia and ischemia from strangulation, alongside imaging and biopsy findings, support the hypothesis of a secondary injury mechanism culminating in DPHL (Fig. 3).

## Why did the patient develop dementia?

The state of unconsciousness in the patient, leading to dementia, can be caused by multiple factors at the brain level. A simplified explanation attributes the patient's dementia to blood flow interruption during strangulation, but this underestimates the cerebral processes involved. One cause could be ischemia, as reduced blood flow leads to excitotoxicity and neuronal damage. Hypoxic-ischemic processes cause neuroinflammation and axonal loss, with demyelination being a hallmark [1, 8]. Diffuse axonal damage is associated with cognitive disorders and behavioral changes [2, 9]. Damage to these areas can directly cause neuropsychiatric problems and dementia. In this patient's case, the loss of consciousness during strangulation initiated acute HIE, followed by DPHL, leading to significant damage to brain structures essential for maintaining consciousness.

## Recommendations in the event of an attack of this magnitude

The patient had multiple predisposing factors contributing to DPHL. First, hypoxia and ischemia are caused by strangulation. This can cause damage to brain tissues, particularly the hippocampus. The delay in receiving medical attention exacerbated the neurological injury. Reoxygenation likely contributed to oxidative stress and further cell damage. Underlying health conditions, like undiagnosed cardiovascular issues, may have worsened the effects of the hypoxic event. These factors together led to DPHL.

A limitation of our case was the opportunity to perform carotid Doppler USG, a study that would allow us to know the initial local repercussions and to have a point of comparison to identify permanent or late damage associated with blood flow alterations. This practice is recommended when blood circulation alterations compromise the cerebral blood flow [10].

## Data Availability

No datasets were generated or analysed during the current study.
